# “Learn from Errors”: Post-traumatic growth among second victims

**DOI:** 10.1186/s12889-024-19738-6

**Published:** 2024-08-28

**Authors:** Huanhuan Huang, Tong Liu, Ying Peng, Xingyao Du, Qi Huang, Qinghua Zhao, Mingzhao Xiao, Yetao Luo, Shuangjiang Zheng

**Affiliations:** 1 Department of Nursing, The First Affiliated Hospital of Chongqing Medical University, Chongqing, 400016 China; 2 School of Public Health, Chongqing Medical University, Chongqing, 400016 China; 3 Department of Urology, The First Affiliated Hospital of Chongqing Medical University, Chongqing, 400016 China; 4 Department of Nosocomial Infection Control, Second Affiliated Hospital, Army Medical University, Chongqing, 400037 China; 5 Department of Medical Affairs, The First Affiliated Hospital of Chongqing Medical University, Chongqing, 400016 China

**Keywords:** Second victim, Patient safety, Post-traumatic growth, Social support, Positive coping

## Abstract

**Background:**

Second victims, defined as healthcare providers enduring emotional and psychological distress after patient safety incidents (PSIs). The potential for positive transformation through these experiences is underexplored but is essential for fostering a culture of error learning and enhancing patient care.

**Objective:**

To explore the level and determinants of post-traumatic growth (PTG), applying the stress process model.

**Methods:**

The study was conducted at a tertiary general hospital in Chongqing, China. A descriptive, cross-sectional study design was used. A total of 474 s victims were included. An online survey was conducted in November 2021 to assess various factors related to the second victim experience. These factors included PSIs (considered as stressors), coping styles, perceived threats, and social support (acting as mediators), as well as the outcomes of second victim syndrome (SVS) and PTG. Statistical description, correlation analysis, and structural equation modeling were utilized for the data analysis. A p-value ≤ 0.05 was considered to indicate statistical significance.

**Results:**

The participants reported moderate distress (SVS = 2.84 ± 0.85) and PTG (2.72 ± 0.85). The total effects on SVS of perceived threat, negative coping, social support, positive coping, and PSIs were 0.387, 0.359, -0.355, -0.220, and 0.115, respectively, accounting for 47% of the variation in SVS. The total effects of social support, positive coping, and PSIs on PTG were 0.355, 0.203, and − 0.053, respectively, accounting for 19% of the variation in PTG.

**Conclusions:**

The study provides novel insights into the complex interplay between perceived threats, coping styles, and social support in facilitating PTG among second victims. By bolstering social support and promoting adaptive coping strategies, the adverse effects of PSIs can be mitigated, transforming them into opportunities for resilience and growth, and offering a fresh perspective on managing PSIs in healthcare settings.

## Introduction

Patient safety incidents (PSIs) not only affect patients and their families but also impact healthcare workers, who may experience adverse psychological consequences [1]. The term ‘second victim’, introduced by Wu, encapsulates healthcare providers affected by unexpected PSIs [2]. Studies indicate that a significant proportion of healthcare providers, ranging from 10.4 to 43.3%, have endured the ordeal of becoming a second victim during their careers [3]. Amidst the COVID-19 pandemic, the well-being of healthcare workers has been thrust into the spotlight, underscoring the importance of safeguarding their well-being for the broader goal of patient safety [4, 5].

Currently, interest in the phenomenon of the second victim continues to grow, with a consensus that these individuals may suffer negative outcomes post-PSIs. They can exhibit a spectrum of psychological and psychosomatic symptoms, including distressing memories, guilt, and sleep disturbances [6–8], presenting significant challenges for the human resources department within the health care system. However, constructivist perspectives suggest that second victims are not merely passive recipients of trauma; instead, they actively interpret their experiences and derive lessons from them [9], potentially leading to posttraumatic growth (PTG) [10].

PTG refers to the process of discovering benefits, promoting stress-related growth, and thriving following a traumatic event [11]. For example, some general surgeons reportedly improved their theoretical and practical knowledge following bile duct injuries during laparoscopic cholecystectomy [12]. Similarly, several studies have reported that second victims often learn valuable lessons from their unexpected and unfortunate clinical experiences, viewing them as part of the natural recovery process [9, 13–15]. It is reported that exploring the lessons learned by second victims addresses their immediate psychological needs and refines the vulnerabilities within patient safety systems [16]. While previous studies have provided valuable insights into the positive outcomes that can arise from critical incidents, there is a significant gap in research that applies a theoretical framework to systematically assess the levels of PTG and its determinants.

The Stress Process Model (SPM), introduced by Pearlin in 1981 [17], is a foundational framework for understanding the dynamics of workplace stress. The model is structured around three core elements: stressors, which are the initiating events; mediators, which influence the stress response; and outcomes, which represent the consequences of stress. SPM provides a systematic approach to analyzing the complex interplay among these elements, making it a valuable tool in various stress-related research domains. SPM has been successfully employed to comprehend phenomena such as depression and burnout in biomedical students [18] and the positive aspects of caregiver burden in dementia [19]. While the SPM has not been extensively applied to patient safety, its conceptual framework resonates with the stress experienced by second victims.

In this study, we defined “outcomes” as two related but distinct adverse consequences of stress, PTG and second victim syndrome. A stressor refers to stimulate that compels individuals to adapt. Previous research has shown that PSIs, such as the degree, type, and frequency of harm, are significant factors in the symptoms experienced by second victims [20]. These factors also play a crucial role in PTG [21].

Mediators refers to the physiological and psychological responses that constitute the perception of being stressed. According to the qualitative research [9, 13–15], mediators may be associated with coping styles, situational influences such as social support, and appraisals (such as perceptions of threat and levels of distress). These mediating factors establish a complex pathway linking stressors and PTG. To elaborate:

(1) Coping strategies are pivotal in shaping an individual’s stress response. Positive methodologies, such as problem solving and social support seeking, can attenuate stress reactions, foster resilience and potentially culminate in PTG [22]. In stark contrast, strategies characterized by avoidance and denial, often categorized as negative, can exacerbate stress responses, impede recovery and potentially lead to the manifestation of second victim syndrome [23].

(2) Perceived threat, a subjective evaluation of the stressor’s potential harm, also plays a crucial role in shaping stress outcomes. A heightened sense of threat can intensify stress reactions, impede recovery, and potentially result in negative outcomes. On the other hand, a diminished perception of threat can moderate stress responses, facilitating adaptation and recovery, and may lead to PTG.

(3) Social support, another key mediator, is characterized by a sense of belonging and attachment to friends, family, or colleagues and includes both emotional and physical support [24]. The ability to connect with others is a critical aspect of PTG [25, 26]. Evidence suggests that a supportive social network, particularly support from managers, can mitigate second-victim syndrome [15, 27, 28]. Recent research has further confirmed that both positive coping styles [29] and perceived threat [30] act as mediators between perceived social support and PTG.

**Fig. 1:**
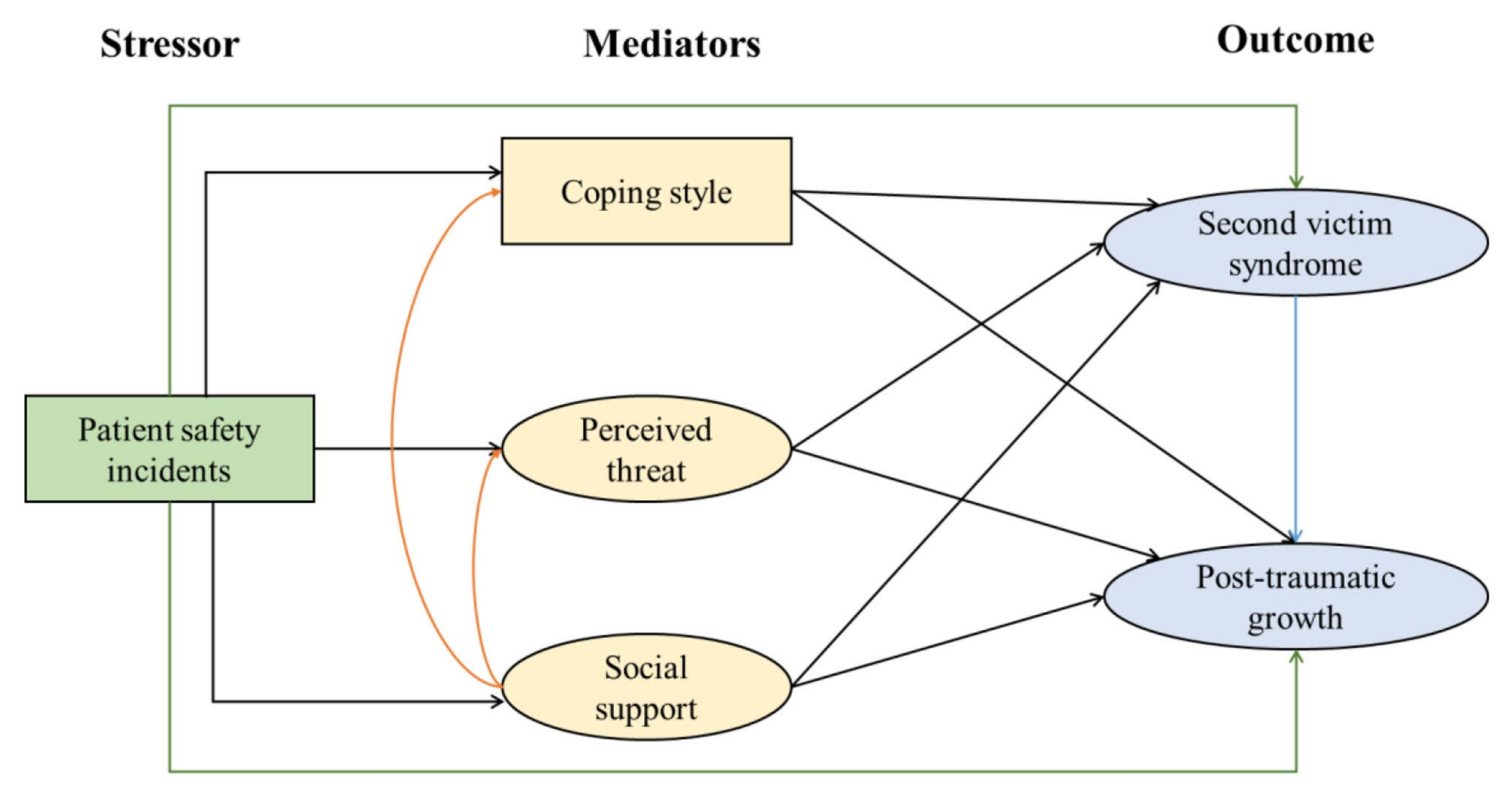
Hypotheses of posttraumatic growth among second victims based on the stress process model

Learning from errors is fundamental to improving patient safety [31]. Thus, this study aimed to (1) assess the level of PTG among second victims, and (2) identify the factors that influence PTG through SPM. The hypothetical model is shown in Fig. 1. Our findings could aid health care systems worldwide in better supporting and understanding these second victims, thereby improving health care provision and patient safety.

## Methods

### Design

We used a descriptive, cross-sectional study design with cluster sampling. The current study utilized the Strengthening Reporting of Observational Studies in Epidemiology (STROBE) cross-sectional checklist [32].

### Setting

This study was conducted in a large, comprehensive hospital, which is a major health care provider in the region. The hospital comprises 38 clinical departments and 8 medical technology departments, with a total of 4616 beds. In 2023, the hospital served an impressive 3.4564 million outpatient visits, discharged 197,100 inpatients, and performed 89,000 surgeries. The patient population is diverse, with individuals coming from all over the country. This diversity in the patient population also translates to a wide range of cases and experiences for health care professionals, thereby adding to the richness and complexity of the data collected in our study.

### Participants

#### Inclusion and exclusion criteria

Drawing upon prior studies [33] and definitions [2], the participants, namely, the second victims, are identified as follows: (a) health care professionals who are directly engaged in patient care or patient management; (b) those who have experienced or witnessed a patient safety incident within the past year; (c) individuals who self-assess as having been negatively impacted during these events; and (d) those who have provided informed consent and demonstrated a willingness to participate.

Our study aimed to capture the experiences of those with a greater degree of responsibility and autonomy in their roles. Given the limited experience of interns and trainees and the supervised nature of their roles, they may not engage with or respond to these incidents in the same depth as our target population. Therefore, interns and trainees were excluded from the study.

### Sample size

The sample size was calculated with a confidence interval of 95% at a proportion of second victims of 76.88% [33], a marginal error of 5%, and a permissible error of 5%. Considering a loss to follow-up rate of approximately 30%, the required sample size was 357. To ensure the reliability of SEM, a priori power analysis based on the recommendation of Kline et al. [34] of a 20:1 sample size-to-parameter ratio was used for estimating the sample size. Based on a maximum of 20 parameters, the priori-determined sample size was 400.

### Measures

#### Patient safety incidents (PSIs)

PSIs are primarily evaluated based on their frequency and severity. First, participants were asked to report the frequency with which they had experienced or witnessed PSIs in the past month. The responses were scored as follows: 3 (always, ≥ 11), 2 (often, 5 ~ 10), 1 (seldom, 1 ~ 5), or 0 (none). Second, participants were required to indicate the type of their most recent PSI based on the definition and classification provided by the Chinese National Health Commission for medical adverse events [35]. The categories were defined as follows: Level I (events with factual errors leading to consequences), Level II (events without factual errors but still leading to significant consequences), Level III (events without factual errors but leading to minor or no consequences), and Level IV (events without factual errors and without any resulting consequences). A cumulative score was calculated based on these two parameters. A higher total score suggests a greater level of stress induced by the PSIs. The reliability of this scoring system was confirmed with a Cronbach’s alpha of 0.712 in this study.

#### Perceived threat

The Perceived Threat Scale (PTS), molded after the Perceived Life Threat Scale [36], was employed to assess the perceived threat. This 4-item tool assessed the perceived threat of PSIs to one’s life or work, the likelihood of similar future incidents, the potential disruption to work or life, and the severity of the incident’s consequences. A 5-point Likert scale was used to quantify the responses. The scale demonstrated acceptable reliability in this study, with a Cronbach’s alpha of 0.756.

#### Coping style

The coping style was assessed using the Trait Coping Style Questionnaire (TCSQ), which is divided into two dimensions: negative coping (NC) and positive coping (PC). Each dimension contains 10 items, and a 5-point Likert scale was used for scoring (5 points for “strongly agree” and 1 point for “strongly disagree”). A higher score in each dimension indicates more pronounced positive or negative strategies. The TCSQ is widely used in the Chinese population, and the two dimensions are typically analyzed separately [37]. In this study, the Cronbach’s alpha was 0.814 for the positive coping dimension and 0.805 for the NC dimension, demonstrating good reliability.

#### Social support

Social support was measured using the Second Victim Experience and Support Tool (SVEST). The SVEST was developed by Burlison et al. [38] and is the first tool designed to evaluate the experiences of second victims and the quality of support resources available to them. This tool has been widely used in China [13, 33]. The social support part includes five dimensions and 18 items, with a Cronbach’s alpha of 0.854, indicating good reliability. The responses are scored using a 5-point Likert scale (5 points for “strongly agree” and 1 point for “strongly disagree”). A higher score on this scale indicates a greater level of perceived social support.

#### Second-victim symptoms (SVS)

The negative outcome, second victim symptoms (SVS), was also assessed using the SVEST of [38]. This aspect of the tool includes two dimensions and eight items, with a Cronbach’s alpha of 0.901, indicating excellent reliability.

#### Posttraumatic growth (PTG)

PTG was measured using the Chinese Posttraumatic Growth Inventory (C-PTGI). The C-PTGI was adapted and translated into Mandarin by Wang [39] from the Posttraumatic Growth Inventory [40]. The C-PTGI consists of 20 items distributed across 5 dimensions. Responses are scored on a six-point Likert scale, with 6 points given for “very much” and 1 point for “not at all”. Based on the participants’ scores, they were categorized into three levels of growth: low (less than 60 points), middle (60–65 points), and high (66–100 points). In this study, the C-PTGI demonstrated excellent reliability, with a Cronbach’s alpha of 0.953.

### Data collection

The online survey was created through a free website (https://www.wjx.cn/). In November 2021, the survey link was disseminated to qualified participants via WeChat groups. This distribution was facilitated by department heads and head nurses to ensure that the link reached the intended audience. The groups were chosen based on the cluster sampling method; each group represented a ‘cluster.’ The clusters were formed based on the departments, specifically including medical departments, surgery departments, and technology-related departments. We carried out the survey across all regular employee within these departments, encompassing three professional categories: nurses, doctors, or medical technical staff. To ensure data quality, we excluded the following questionnaires: (a) those with identical answers on both reverse and forward questions; (b) those completed in less than nine minutes, as per the pilot study; and (c) those in which all of the answers were the same.

### Data analysis

Data analysis was performed using IBM SPSS and AMOS version 26.0 (IBM Corp, Armonk, NY, USA). We used descriptive statistics to describe the demographic information. Continuous data, such as the scores from the C-PTGI, are reported as the means along with their standard deviations (SDs). To ensure the reliability of our model, we first checked for multicollinearity among the independent variables by calculating the variance inflation factor (VIF). The results showed that the VIF for all variables was less than 3, indicating no significant multicollinearity issues among the variables. Bivariate analyses were conducted to explore the relationships among all of the variables. To identify the underlying mechanisms of PTG among second victims, we employed structural equation modeling (SEM). We assessed the model fit using several indices [41]: a relative chi-square (χ2/df) less than 5, a goodness-of-fit index (GFI) over 0.9, a comparative fit index (CFI) above 0.90, and a root mean squared error of approximation (RMSEA) below 0.08. The bootstrap method (with 5000 samples) was used to calculate the 95% confidence intervals (CIs) for direct effects, indirect effects, and total effects. A P value less than 0.05 (two-sided) was considered to indicate statistical significance.

### Ethical consideration

Ethical approval for the study was obtained from the Ethics Committee of the First Affiliated Hospital of Chongqing Medical University (reference number 2019-067). Participants were assured that their decision to participate or not would not affect their professional status or opportunities within the institution. Only those who provided written consent on the first page of the survey were allowed to proceed with answering the questions. They were informed that they could withdraw from the study at any time. To ensure confidentiality, any data sharing or reporting was conducted in a manner that protected the privacy of individual participants. To prevent any unnecessary administrative coercion and to ensure the integrity of the data, each questionnaire was anonymized. Additionally, measures were taken to permit each IP address to submit the survey only once.

## Results

### Demographic characteristics of the participants

Ultimately, a total of 1023 health care service providers actively participated and completed the survey, 238 of whom were excluded due to inadequate quality. Furthermore, an additional 311 respondents were excluded because they did not meet the criteria for being considered second victims. Consequently, a final analysis was conducted utilizing data from 474 eligible participants.

Most participants were female (75.11%) and married (80.80%). Approximately half of the participants were nurses (51.27%), with ages ranging from 31 to 40 years (58.64%). Of these, 42.83% worked in medical departments, while 85% worked in surgery departments; only a minority of participants worked in technology-related departments such as radiology departments (20%). Table 1 presents the demographic characteristics of all participants.

**Table 1:** Demographic characteristics of the participants (*n* = 474)

Characteristics	*N*	%
Sex		
Male	118	24.89
Female	356	75.11
Age (years)		
21–30	86	18.14
31–40	278	58.65
41–50	71	14.98
51–60	39	8.23
Occupation		
Nurse	243	51.27
Doctor	171	36.08
Medical technical staff	60	12.66
Departments		
Medical departments	203	42.83
Surgery departments	195	41.14
Technology-related departments	76	16.03
Education level		
College degree	17	3.59
Bachelor degree	244	51.48
Master degree or above	213	44.94
Marriage statues		
Married	383	80.80
Unmarried	91	19.20
Work experience (years)		
≤5	85	17.93
6–10	158	33.33
11–20	151	31.86
> 20	80	16.88

### Descriptive statistics and correlations of the variables

The mean PSIs score was 1.80 (SD = 0.54). Participants reported a slight perceived threat (mean = 2.19, SD = 0.81). Coping style scores indicated a greater mean for PCs (3.17 ± 0.54) than for NCs (2.71 ± 0.56). Participants displayed a high level of social support (3.67 ± 0.53) and moderate distress (SVS = 2.84 ± 0.85). The mean C-PTGI score was 2.72 ± 0.85, indicating a moderate level of growth, with the highest score for personal strength (3.08 ± 0.99) and the lowest for spiritual change (2.59 ± 0.97). According to the C-PTGI cutoff, 36.06% of second victims reported some level of psychological improvement after PSIs. PTG had negative and positive associations with NC (*r*=-0.190, *p* < 0.001) and perceived threat, PC, and social support (*r* = 0.098, *r* = 0.288, *r* = 0.354; all *p* < 0.05), respectively. Details were shown in Table 2.

**Table 2:** Pearson correlation coefficients for the study variables

Variable	Mean ± SD	1	2	3	4	5	6
1. patient safety incidents	1.80 ± 0.54	1					
2. perceived threat	2.19 ± 0.81	0.216***	1				
3. positive coping	3.17 ± 0.54	-0.153***	-0.109*	1			
4. negative coping	2.71 ± 0.56	0.117*	0.225***	-0.494***	1		
5. social support	3.67 ± 0.53	-0.112*	-0.221***	0.335***	-0.329***	1	
6. second victim’s symptoms	2.84 ± 0.85	0.142**	0.353***	-0.375***	0.503***	-0.270***	1
7. post-traumatic growth	2.72 ± 0.85	-0.035	0.098*	0.288***	-0.190***	0.354***	-0.013

SD: Standard deviation

*P < 0.05, **P < 0.01, *** *P* < 0.001

### Path analysis results

**Fig. 2:**
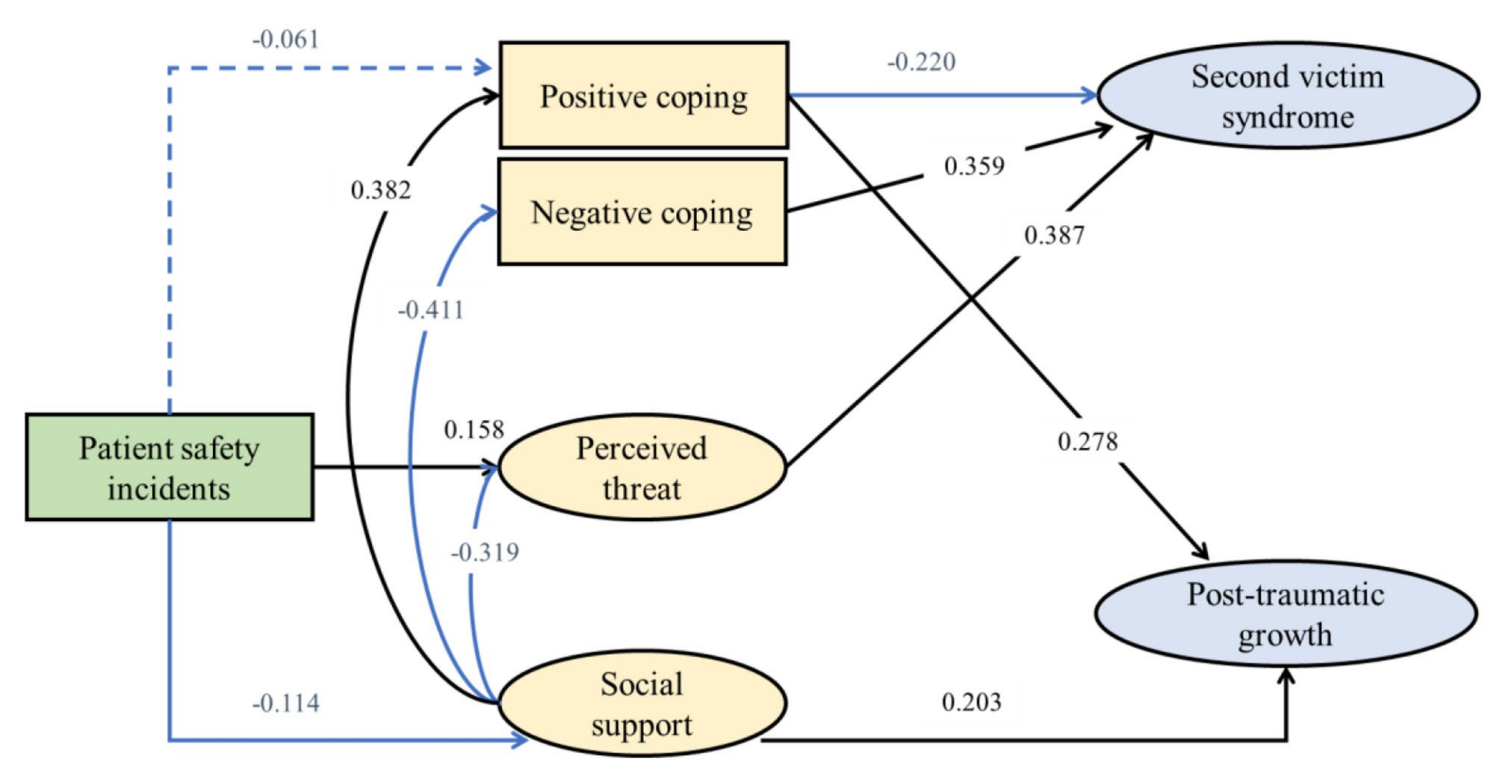
The final model for factors influencing post-traumatic growth in second victims. (with standardized regression coefficients). *p < 0.05; **p < 0.01; ***p < 0.001

The initial model results showed an unsatisfactory fit, leading to model modification. Nonsignificant paths (PSIs → NC, PSIs → SVS, social support → SVS, PSIs → PTG, NC → PTG) and paths inconsistent with the correlation analysis results (SVS → PTG, perceived threat → PTG) were removed. The final model demonstrated a good fit (χ2/df = 365.614/199 = 3.072, GFI = 0.922, CFI = 0.937, and RMSEA = 0.066). The total path estimates for the final model are shown in Table 3, and the validated model with standardized effects among variables is depicted in Fig. 2.

### Direct effect

PC (direct β=-0.220, 95% CI=-0.328~ -0.115), NC (direct β=-0.359, 95% CI = 0.237 ~ 0.478), and perceived threat (direct β = 0.387, 95% CI = 0.281 ~ 0.493) had direct effects on SVS. Social support (direct β = 0.278, 95% CI = 0.148 ~ 0.404) and PC (direct β = 0.203, 95% CI = 0.108 ~ 0.295) directly influenced PTG.

### Direct effect

PSIs positively influenced SVS indirectly via social support (indirect β = 0.041, 95% CI = 0.004 ~ 0.082) and perceived threat (indirect β = 0.061, 95% CI = 0.022 ~ 0.11). PSIs had a negative indirect effect on PTG primarily through social support (indirect β=-0.041, 95% CI=-0.084~-0.006). Social support indirectly influenced SVS negatively through PC (indirect β=-0.084, 95% CI=-0.138~-0.041), NC (indirect β=-0.148, 95% CI=-0.213~-0.097) and perceived threat (indirect β=-0.124, 95% CI=-0.186~-0.072). Its positive indirect effect on PTG was primarily via social support (indirect β = 0.078, 95% CI = 0.042 ~ 0.125).

### Total effect

The total effects on SVS of perceived threat, NC, social support, PC, and PSIs were 0.387, 0.359, -0.355, -0.220, and 0.115, respectively, accounting for 47% of the variation in SVS. The total effects of social support, PC, and PSIs on PTG were 0.355, 0.203, and − 0.053, respectively, accounting for 19% of the variation in PTG.

**Table 3:** The total path estimates in the final model

Path	β	Bootstrap method 95% CI ^a^		
		Lower	Upper	*P*
**Direct effect**				
PSIs →PC	-0.061	-0.136	0.014	0.117
PSIs →Perceived Threat	0.158	0.055	0.264	0.003
PSIs →Social Support	-0.114	-0.215	-0.009	0.029
Social Support →PC	0.382	0.279	0.473	< 0.001
Social Support →NC	-0.411	-0.504	-0.316	< 0.001
Social Support → Perceived Threat	-0.319	-0.438	-0.195	< 0.001
PC → SVS	-0.220	-0.328	-0.115	< 0.001
NC →SVS	0.359	0.237	0.478	< 0.001
Perceived Threat →SVS	0.387	0.281	0.493	< 0.001
Social Support →PTG	0.278	0.148	0.404	< 0.001
PC →PTG	0.203	0.108	0.295	< 0.001
**Indirect effect**				
PSIs →Social Support →PC→SVS	0.010	0.002	0.023	0.015
PSIs →Social Support →NC→SVS	0.017	0.002	0.038	0.021
PSIs →Social Support →Perceived Threat →SVS	0.014	0.002	0.032	0.019
PSIs →Social Support →SVS	0.041	0.004	0.082	0.025
PSIs →PC→SVS	0.013	-0.001	0.034	0.073
PSIs →Perceived Threat →SVS	0.061	0.022	0.110	0.002
PSIs →SVS	0.115	0.062	0.176	< 0.001
Social Support→ PC→SVS	-0.084	-0.138	-0.041	< 0.001
Social Support →NC→SVS	-0.148	-0.213	-0.097	< 0.001
Social Support →Perceived Threat →SVS	-0.124	-0.186	-0.072	< 0.001
Social Support →SVS	-0.355	-0.435	-0.275	< 0.001
PSIs →Social Support →PTG	-0.032	-0.072	-0.005	0.017
PSIs →Social Support →PC→PTG	-0.009	-0.021	-0.002	0.014
PSIs →Social Support →PTG	-0.041	-0.084	-0.006	0.021
PSIs →PC→PTG	-0.012	-0.033	0.002	0.089
PSIs →PTG	-0.053	-0.096	-0.014	0.010
Social Support →PC →PTG	0.078	0.042	0.125	< 0.001
**Total effect**				
PSIs →SVS	0.115	0.062	0.176	< 0.001
Social Support →SVS	-0.355	-0.435	-0.275	< 0.001
PC → SVS	-0.220	-0.328	-0.115	< 0.001
NC →SVS	0.359	0.237	0.478	< 0.001
Perceived Threat →SVS	0.387	0.281	0.493	< 0.001
PSIs →PTG	-0.053	-0.096	-0.014	0.010
Social Support →PTG	0.355	0.234	0.471	< 0.001
PC → PTG	0.203	0.108	0.295	< 0.001

PSIs: Patient safety incidents; PTG: Post-traumatic growth

β: Standardized regression coefficient; SE: Standard error

^a^ The bootstrap method yielded 5000 samples

## Discussion

To the best of our knowledge, this is the first study investigating the underlying mechanisms of SVS and PTG among second victims. The final model indicated that perceived threats, coping styles, and social support as stressors significantly influenced the outcomes. These findings have important implications for understanding and responding to the effects on second victims, revealing the important role of coping strategies and social support in the aftermath of patient safety incidents.

Our research revealed that second victims exhibit moderate levels of SVS and PTG. This finding aligns with previous studies on survivors of different types of trauma [42, 43] and conforms to the fundamental concept of PTG, which posits that the positive and negative aspects of adjustment are independent [44]. This suggests that persistent distress and growth are not mutually exclusive and can coexist within an individual, a notion that is consistent with constructivism and positive psychology [45, 46]. In our study, we found that the average PTG score for the personal strength dimension was high, whereas the average PTG score for the spiritual change dimension was low. The medical profession has a long-standing commitment to continuing education, ensuring that all health care providers and students maintain the highest quality of care [47]. This emphasis on continuous learning is particularly notable among those who have been victims or witnesses of adverse events or PSIs. Occasionally, the ‘shame and blame’ culture or administrative policy within institutions has even served as a catalyst for the advancement of theory and practice improvement for second victims [48, 49]. On the other hand, psychological growth was found to be relatively low. Only 36.06% of second victims reported experiencing positive growth following patient safety incidents, a finding that aligns with previous studies [50] and warrants further attention and consideration.

Our study revealed that only PC and social support had direct effects on PTG, with total effects of 0.355 and 0.203, respectively. Furthermore, our results partially supported our hypotheses and previous findings [51–53], indicating that social support indirectly influences PTG through the style of PC. This implies that second victims with higher levels of social support are likely to receive more emotional or instrumental support, which may help them reshape their perception of errors and cope with the outcomes. This finding is consistent with other studies in which second victims reported that unsupportive practices could hinder the restoration of professional competence, thereby preventing them from “moving forward“ [54]. This suggests that in designing and implementing interventions, we need to pay more attention to how to provide effective social support and how to foster positive coping mechanisms. Indeed, these two elements are also highlighted in second victim support programs, such as the forYOU [55] and RISE (Resilience in Stressful Events) peer support programs [56].

Our study elucidates intriguing findings that question conventional paradigms regarding the relationship between trauma and ensuing growth. Specifically, contrary to our hypothesis, we observed that PSIs, perceived threats, negative coping strategies, and SVS did not facilitate PTG. The absence of this expected relationship might be attributable to the inherent characteristics of PSIs, which frequently precipitate substantial negative ramifications and engender feelings of culpability rather than fostering opportunities for growth [57]. This could be partly explained by the existence of an inverted-U shaped relationship between distress levels and PTG, whereby both excessively high and low distress levels obstruct the perception of growth [58]. Such a revelation necessitates a comprehensive reevaluation of our understanding of PTG, highlighting the potential merit of shifting our focus toward factors that encourage positive coping and adaptation, as opposed to concentrating exclusively on the trauma itself.

Our research provides a fresh understanding of PTG among second victims. The findings suggest that PTG in second victims is not attributed to the suffering experienced or the threats they perceived, but rather a proactive response, tool support, and active refactoring [51]. This insight underscores that learning from errors is not just about experiencing distress but also about how to positively process and learn from these experiences, a viewpoint corroborated by previous research [59, 60]. For instance, studies have shown that proactive rumination can help individuals positively interpret and deal with traumatic events, overcome fear responses, and foster constructive responses, thereby supporting their growth post-trauma [42]. In contrast, intrusive rumination leads individuals to negatively interpret traumatic events and focus on their negative aspects, causing more anxiety and tension and potentially exacerbating psychological trauma [42]. Thus, our results highlight the importance of positive coping and social support in promoting PTG in second victims.

Notably, our final model for second victims’ PTG explained only a small amount of variance (19%), which may be attributed to potentially unrecognized confounding factors. As an adaptive system, health care delivery involves complexity and uncertainty [61, 62] as agents, and the relationships between these agents is constantly evolving. Thus, it is challenging to fully comprehend the entire system. Given this characteristic, perceived growth after adverse events or errors may emerge from a confluence of numerous contributory factors.

Overall, our findings have broad implications for future research and provide directions for hospital human resource management and quality improvements. The safety of health care workers is a prerequisite for patient safety. Investing in support for second victims should be a priority for patient safety. Although errors are inevitable, health care managers should formulate practical strategies and provide available resources to assist second victims. Implementing specific strategies, such as group interventions, can be beneficial, allowing second victims to share their experiences and gain strength from the experiences and support of others [63]. Mindfulness training could also be helpful [64], as it can assist second victims in better processing and accepting their experiences rather than avoiding or resisting them.

### Limitations

As with any study, it is important to consider the limitations of this study when drawing conclusions. First, this cross-sectional design study made it difficult to infer cause-and-effect relationships. More long-term cohort studies are needed to fully confirm this hypothesis. This setting, with its large scale and diverse patient pool, provides a unique and comprehensive environment for our study of second victims, despite the limitation of being a single-site study. Third, the use of a self-report questionnaire may lead to reporting bias and socially desirable responses. Finally, some other factors may explain the positive psychological changes, which could also cause bias.

## Conclusions

Our cross-sectional research provides a new understanding of the intricate relationships among perceived threats, coping mechanisms, and social support in the context of PTG. By strengthening social support and enhancing adaptive coping strategies, we can shift the consequences of an error from trauma to resilience and growth, offering a fresh approach to managing the repercussions of patient safety incidents.

## Data Availability

The data and materials are available from the corresponding author.
